# Efficacy of music therapy on mild cognitive impairment in the aging population: A systematic review and meta-analysis

**DOI:** 10.1097/MD.0000000000047299

**Published:** 2026-01-16

**Authors:** Yi Li, Xiaorong Jiang, Meiyu Zhu, Ximei Li, Yu Wang

**Affiliations:** a Health Management Center, Jinjiang Municipal Hospital (Shanghai Sixth People’s Hospital Fujian), Jinjiang, China; b Health Management Center, Jinjiang Municipal Hospital Jinnan Branch, Jinjiang, China.

**Keywords:** aging, mild cognitive impairment, music, music therapy

## Abstract

**Background:**

Mild cognitive impairment represents an intermediate stage between normal aging and dementia, affecting memory, executive function, and daily living in older adults. This meta-analysis was conducted to assess the efficacy of music therapy on cognitive and neuropsychological outcomes in older individuals with mild cognitive impairment.

**Methods:**

Searches of PubMed, EMBASE, Web of Science, China National Knowledge Infrastructure, and Wan Fang databases from inception to December 30, 2024. Standardized mean differences for mini-mental state examination (MMSE) scores, comparing older adults with mild cognitive impairment to control groups, were pooled using a fixed-effect model. Publication bias was assessed by inspecting funnel plot symmetry and conducting Egger regression test.

**Results:**

A total of 8 studies were included in our study. A fixed effects model was used to pool the data because the heterogeneity tests indicated a low degree of heterogeneity (*I*^2^ = 42.6%, *P* = .094). The results indicated an increased MMSE score in the patients with music therapy than the control group (SMD = 0.58; 95% CI: 0.37–0.78; *P* < .001).

Funnel plots and Egger test revealed no significant evidence of publication bias.

**Conclusion:**

This meta‐analysis suggests that music therapy can improve cognitive outcomes in older adults with mild cognitive impairment.

## 1. Introduction

Mild cognitive impairment involves a level of cognitive decline that exceeds what would be expected for an individual’s age and educational background. People with mild cognitive impairment experience difficulties in memory, language, attention, reasoning, or other cognitive domains but remain largely independent in daily activities and do not meet the criteria for dementia.^[1]^ Mild cognitive impairment represents a prodromal stage situated between the age-appropriate cognitive state and the earliest clinical signs of dementia.^[2]^ An estimated 10 to 15 percent of adults aged 65 and older are affected by mild cognitive impairment worldwide.^[3]^ Individuals with mild cognitive impairment face a markedly higher risk of progression to Alzheimer type dementia, with annual conversion rates estimated between 10.2 and 33.6 percent, compared with a 1 to 2% rate in the general older population, which not only places a significant financial burden on healthcare systems but also exacts substantial personal and societal costs.^[4]^ Early recognition and management of mild cognitive impairment are therefore essential to delaying or preventing further cognitive decline.

Over the past 2 decades, the clinical application of music therapy has expanded markedly as a non-pharmacological approach to support cognitive health.^[5]^ Music functions as a complex sensory stimulus that elicits neurophysiological responses in the brain, engaging networks responsible for cognition, motor coordination, and emotional regulation.^[6]^ Through these mechanisms, music therapy has been shown to enhance mental well-being, memory retention, and executive functioning in older adults with mild cognitive impairment.^[7,8]^ Although numerous individual studies have explored these benefits, the evidence remains dispersed and lacks a standardized synthesis. To address this gap and provide a robust, evidence-based foundation for clinical practice, a systematic review and meta-analysis of existing trials was undertaken to quantify the sole listening-based music therapy on mild cognitive impairment.

## 2. Methods

The meta-analysis was conducted in accordance with the guidelines set forth by the Preferred Reporting Items for Systematic Reviews and Meta-Analyses.^[9]^ This study was conducted in accordance with the Helsinki Declaration of 1975 as revised in 2024. All patient details were de-identified. Additionally, this prospective review has been registered with PROSPERO under registration number CRD420251039186.

### 2.1. Search strategy

A systematic literature search was conducted to evaluate the effects of music interventions for mild cognitive impairment. Major databases including PubMed, EMBASE, Web of Science, China National Knowledge Infrastructure, and Wan Fang databases, were searched from their inception to December 30, 2024, without any language restrictions. The search terms were as follows: (“music therapy” [mesh] OR “music” [mesh] OR “auditory stimulation” [mesh] OR “arts therapy” [mesh]) AND (“mild cognitive impairment” [mesh] OR “cognitive dysfunction” [mesh] OR “cognition” [mesh] OR “memory” [mesh] OR “memory disorders” [mesh] OR “dementia” [mesh]).

### 2.2. Inclusion and exclusion criteria

Inclusion criteria: Participants must be adults diagnosed with mild cognitive impairment; The intervention must involve any form of music therapy or music intervention; Studies must report on cognitive or neuropsychological outcomes related to the efficacy of the intervention; and Studies must be published in peer-reviewed journals. Exclusion criteria: Participants younger than 65 years; Case reports, reviews, and animal studies; Studies with significant errors in data or incomplete data records; Duplicate publications or studies for which the full text is inaccessible; Non-Chinese and non-English literature; Studies not specifying a diagnosis of mild cognitive impairment; and Studies involving active modalities (e.g., instrument playing or singing) were excluded.

### 2.3. Data extraction

Literature screening and data extraction were performed independently by 2 researchers. In cases of discrepancies, a third researcher was consulted for resolution. The data extraction process utilized a standardized collection form to ensure consistency and accuracy. Extracted data included essential information such as author, publication year, country, participant age, gender, total intervention duration, frequency, single session duration, and mini-mental state examination (MMSE) scores.

### 2.4. Statistical analysis

Statistical analyses were carried out using Stata 16.0 software. Heterogeneity among the studies was evaluated using the *I*^2^ statistic and Cochran *Q* test. According to the standard meta-analysis conventions, Cochrane Handbook guidance, and the level of heterogeneity observed, 2 analytical models were implemented: a random effects model was used when *P* < .10 and *I*^2^ > 50%, indicating substantial heterogeneity, while a fixed effects model was employed when *P* ≥ .10 and *I*^2^ ≤ 50%, suggesting little to no heterogeneity. Continuous measurement data were reported as standardized mean differences (SMD), with 95% confidence intervals (CIs) reflecting the overall effect size of the meta-analysis. Publication bias was examined through funnel plots, with any asymmetry assessed using the Egger regression test. Additionally, sensitivity analyses (leave-one-out analysis) were performed to investigate how individual studies might influence the overall results.

## 3. Results

### 3.1. Search strategy

**Figure 1.**
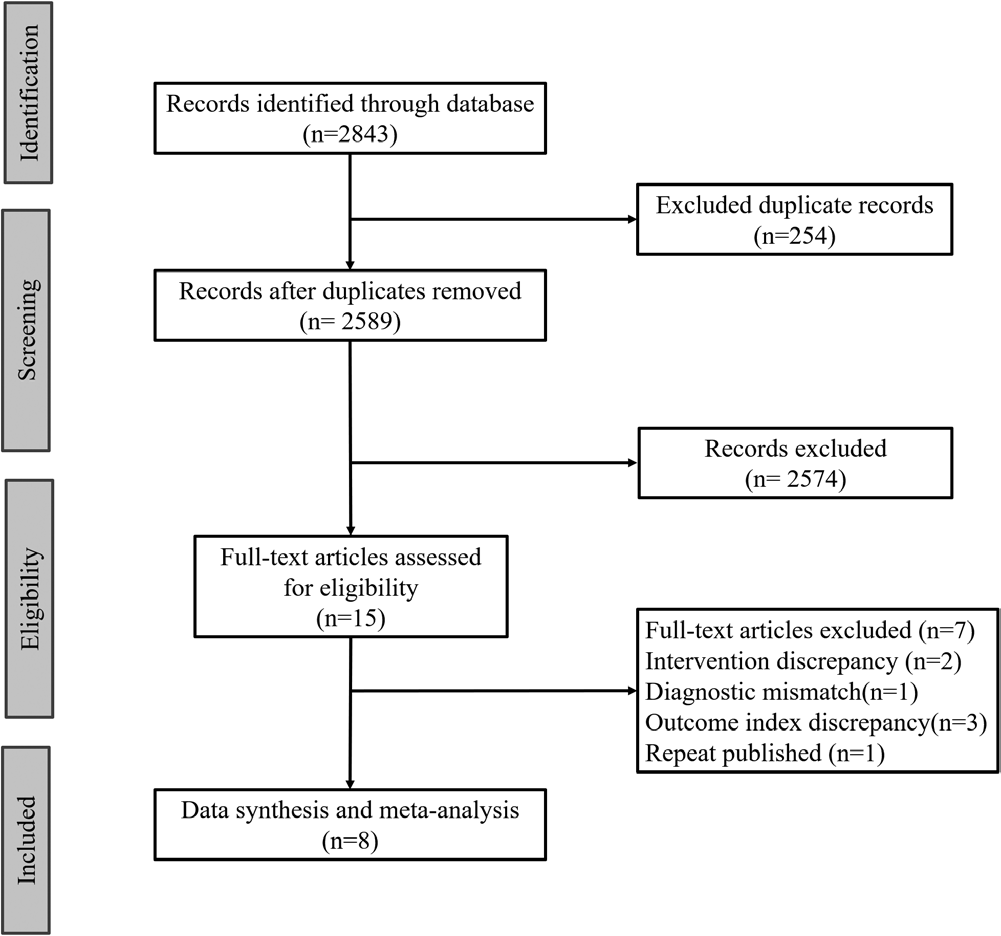
Flowchart of the study selection.

The initial search identified 2843 articles through both electronic and manual database sources. After removing duplicates, 2589 unique studies remained for screening. During the title and abstract review, articles categorized as reviews, case reports, conference abstracts, or irrelevant studies (did not meet the predefined inclusion criteria) were excluded. This process resulted in 15 articles for full-text evaluation. Of these, 7 did not meet the inclusion criteria and were excluded (intervention discrepancy (n = 2), diagnostic mismatch (n = 1), outcome index discrepancy (n = 3), repeat published (n = 1). Ultimately, 8 studies were included in the meta-analysis. The search strategy flowchart is summarized in Figure 1.

### 3.2. Characteristics of included studies

This systematic review and meta-analysis encompassed a total of 381 aging patients who were diagnosed with mild cognitive impairment. As detailed in Table 1, the studies were geographically diverse, including China,^[10,11]^ Japan,^[12,13]^ Korea,^[14,15]^ and Italy.^[16,17]^ A key characteristic of the interventions was their variability in duration. For instance, long-term interventions of 12 months were conducted by Xiong et al^[10]^ and Satoh et al,^[13]^ whereas shorter programs (<6 months) were more common.^[11,15–17]^ The baseline cognitive status, as measured by MMSE scores, also showed a range across the included cohorts.

**Table 1:** Characteristics of included studies.

Study ID	Years	Country	Age	Gender (M/F)	Total time	Frequency	Single duration	MMSE scores	
								Music therapy	Control
Xiong et al^[10]^	2022	China	74.00 ± 6.07	10/20	12 mo	≥7 times per wk	1 h	24.00 ± 2.22	20.00 ± 5.19
Miao et al^[11]^	2020	China	74.03 ± 4.99	32/28	<6 mo	≥7 times per wk	<1 h	23.03 ± 2.33	21.20 ± 1.61
Satoh M et al^[12]^	2017	Japan	87.00 ± 5.40	N/A	6 mo	<5 times a wk	<1 h	20.80 ± 4.40	21.50 ± 5.50
Satoh M et al^[13]^	2014	Japan	72.40 ± 3.70	52/9	12 mo	<5 times a wk	1 h	28.70 ± 1.80	27.20 ± 2.20
Kim H-J et al^[14]^	2016	Korea	78.44 ± 1.00	16/37	6 mo	5 times a wk	1 h	19.28 ± 6.02	15.04 ± 7.00
DeokJu Kim et al^[15]^	2020	Korea	80.60 ± 5.12	9/26	<6 mo	5 times a wk	1 h	19.56 ± 2.17	17.68 ± 1.64
Biasutti M et al^[16]^	2018	Italy	83.39 ± 7.31	13/22	<6 mo	<5 times a wk	>1 h	25.00 ± 3.39	23.47 ± 4.46
Biasutti M et al^[17]^	2021	Italy	89.95 ± 7.84	16/29	<6 mo	<5 times a wk	>1 h	24.75 ± 3.40	22.40 ± 4.73

MMSE = mini-mental state examination.

### 3.3. Quality of the included studies

**Figure 2.**
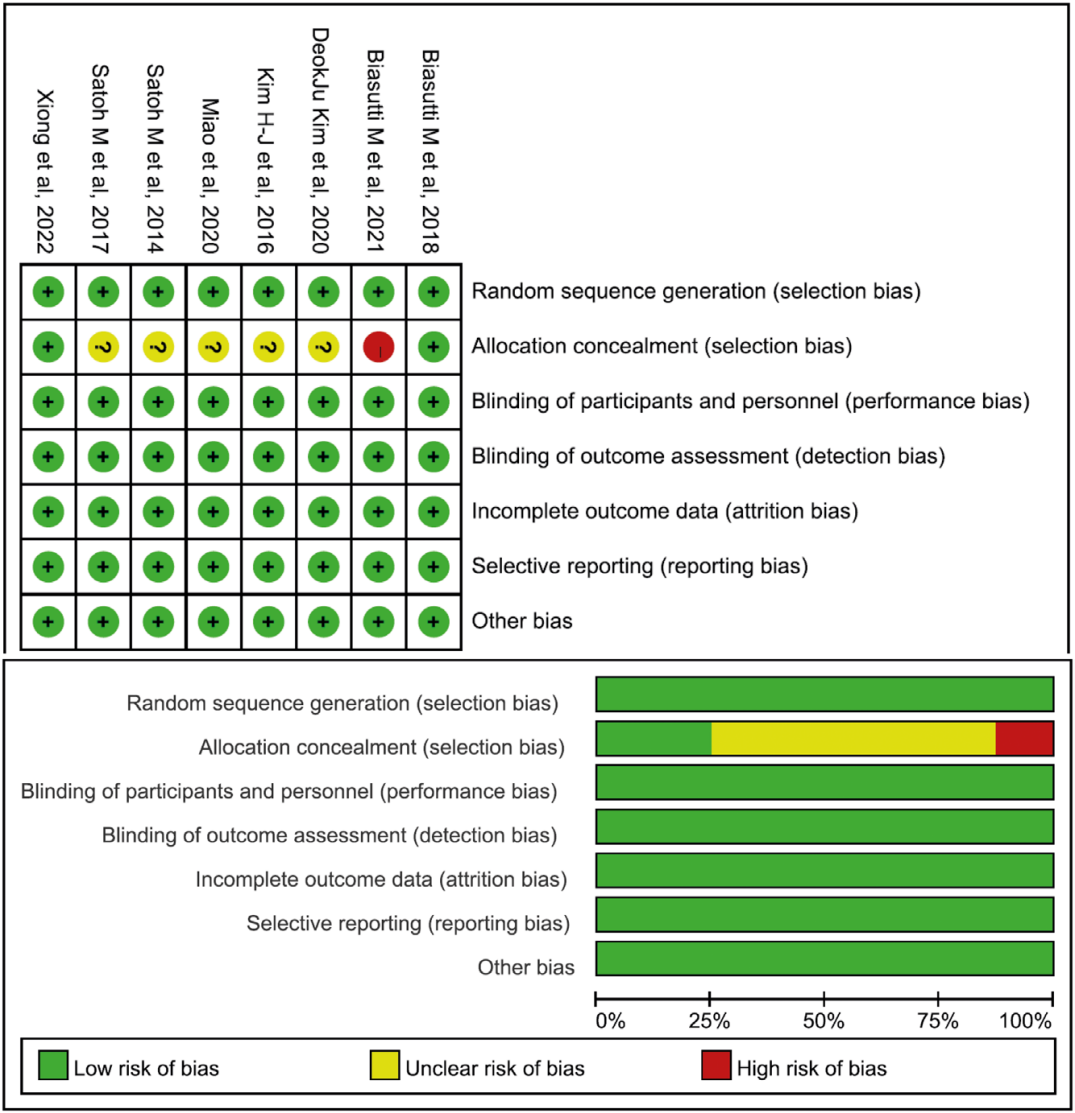
Risk of bias assessment overview: researchers’ assessments for each specific item of ROB 2 for the included study. ROB 2 = risk of bias tool.

The risk of bias was evaluated in all 8 relevant studies (Fig. 2). The assessment revealed that 5 randomized controlled trials presented some concerns regarding the risk of bias, but none of them were at a serious risk of bias. The study by Biasutti M et al^[17]^ was deemed to have induced bias in the allocation concealment of the selection domain.

### 3.4. Results of meta-analysis

**Figure 3.**
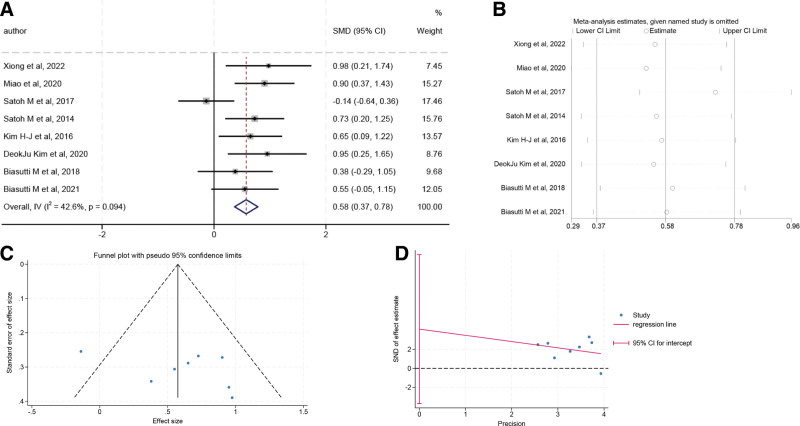
Meta-analysis of the efficacy of music therapy on mild cognitive impairment in the aging population. (A) Forest plot of the effect of music therapy on MMSE scores in individuals with mild cognitive impairment; (B) sensitivity analysis; (C) funnel plot; and (D) Egger linear regression test. MMSE = mini-mental state examination.

In this meta-analysis, 8 studies investigated the variance of MMSE scores in individuals with music therapy and control groups. A fixed effects model was used to pool the data because the heterogeneity tests indicated a low degree of heterogeneity (*I*^2^ = 42.6%, *P* = .094). The results indicated an increased MMSE score in the patients with music therapy than the control group (SMD = 0.58; 95% CI: 0.37–0.78; *P* < .001) (Fig. 3A). A sensitivity analysis was conducted by omitting each study, and the resulting effect estimates remained consistent, confirming the robustness and reliability of the meta-analysis findings (Fig. 3B). Publication bias was assessed by visual inspection of the funnel plot, which showed a symmetric distribution of studies, and by Egger linear regression test (*t* = 1.29, *P* = .245), indicating no evidence of publication bias (Fig. 3C and D).

### 3.5. Subgroup analysis

**Figure 4.**
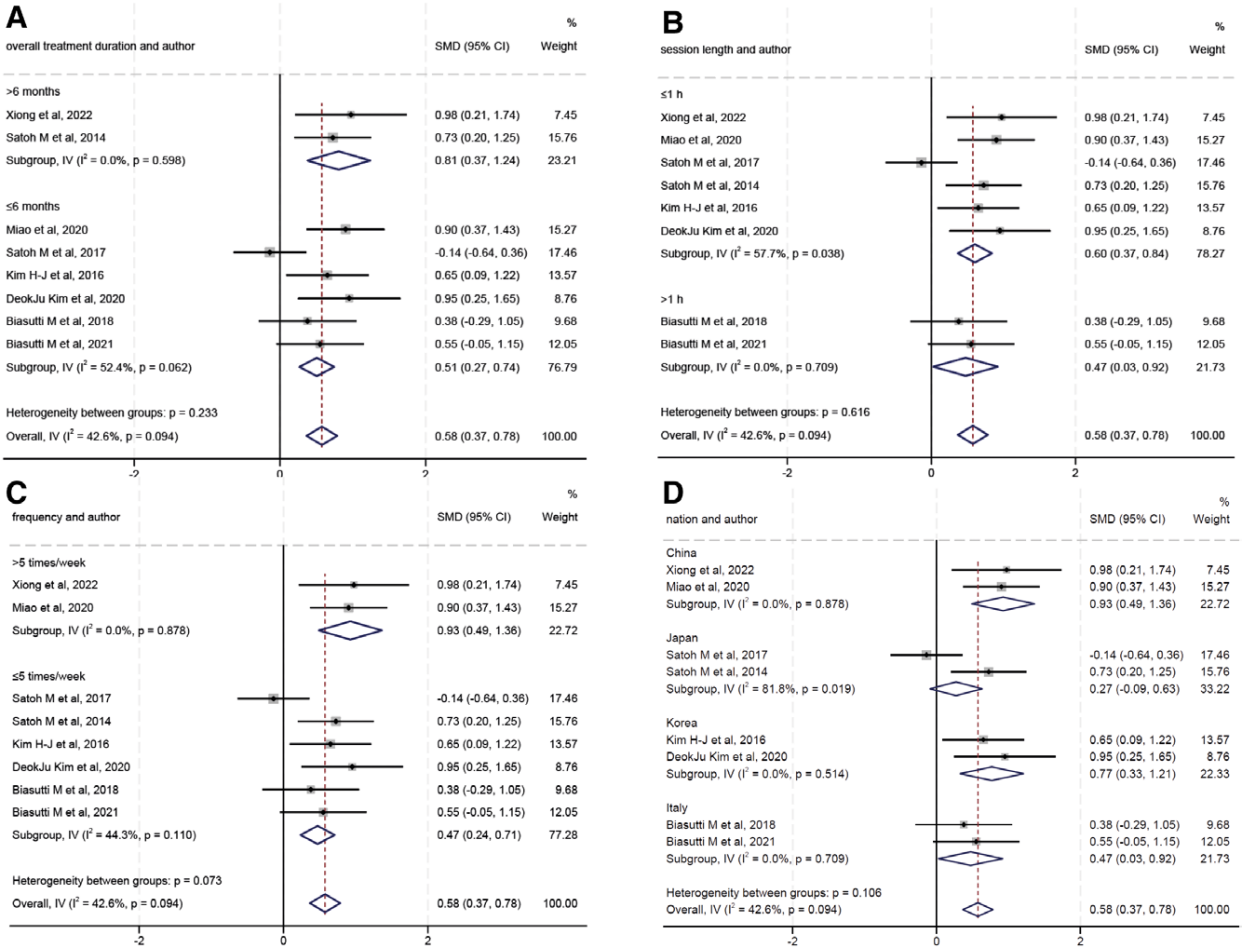
Subgroup analysis of the efficacy of music therapy on mild cognitive impairment in the aging population. (A) Pverall treatment duration; (B) session length; (C) frequency; and (D) country.

Subgroup analyses assessed whether the effects of music therapy on mild cognitive impairment differed by overall treatment duration (≤6 months vs >6 months), session length (≤1 hour vs >1 hour), frequency (≤5 times/week vs >5 times/week), and country. Pooled analysis of the 8 studies showed that music therapy significantly increased the MMSE scores in patients with mild cognitive impairment in both overall treatment duration (≤6 months and >6 months) groups (Fig. 4A). A similar trend was observed in session length (Fig. 3B), frequency (Fig. 4C), and country (Fig. 4D) subgroup analyses.

## 4. Discussion

In this systematic review and meta-analysis, 8 studies comprising 381 older adults with mild cognitive impairment met the inclusion criteria. The pooled findings indicate that music therapy, as a simple and non-drug intervention, produces significant gains in global cognition, reflected by improvements in MMSE scores and observable benefits in memory, orientation, attention, and language functions. However, relying solely on the MMSE limits our ability to detect subtle cognitive changes and behavioral symptoms due to its ceiling effects and relatively low sensitivity. The Montreal Cognitive Assessment offers greater sensitivity to mild deficits and captures cognitive heterogeneity more effectively,^[18]^ while the Neuropsychiatric Inventory systematically evaluates neuropsychiatric symptoms that the MMSE cannot assess.^[19]^ Subgroup analyses, however, were unable to identify an optimal overall treatment duration, session length, or weekly frequency, and variability in intervention protocols precludes definitive recommendations. High-quality, large-scale trials are needed to establish evidence-based guidelines for the “dose” of music therapy in older adults with mild cognitive impairment.

Numerous investigations have shown that music listening can boost learning, memory, and overall cognitive performance in older adults with mild cognitive impairment.^[20]^ Molecular imaging with positron emission tomography, functional magnetic resonance imaging, diffusion tensor imaging, magnetic resonance spectroscopy has been used to examine the alterations in functional connectivity of brain regions in mild cognitive impairment.^[21]^ Neuroimaging research reveals that hearing music strengthens functional connectivity across a broad array of brain regions, including the frontal and parietal cortices, which in turn supports lyric recall, digit‐span capacity, and working memory.^[22]^ Beyond memory enhancement, familiar or preferred tunes further sharpen orientation, attention and executive control by increasing activity in the default mode network and the prefrontal cortex, key nodes for emotion and higher‐order cognition.^[22,23]^ Distinct acoustic elements also recruit specialized circuits: timbre processing engages cerebellar and sensory cortices as well as default network regions, while rhythm and tonality drive both cognitive‐motor loops and emotion‐related pathways.^[24]^ Moreover, chromatic melodies elicit greater activation in areas responsible for hierarchical sequencing, conflict resolution and working memory than diatonic scales.^[25]^ Receptive music therapy exploits these properties by using rhythmic and soothing compositions to induce relaxation and emotional balance, thereby modulating brain‐derived neurotrophic factor expression, preserving hippocampal integrity and integrating autobiographical memory, self‐awareness and communication skills.^[26,27]^ Listening to music also modulates activity in the frontal lobes and limbic regions involved in visual information processing, and both music listening and performance increase expression of brain-derived neurotrophic factor, which can enhance cognitive function and mood.^[28]^

Studies in people with cognitive impairment show that listening to familiar and preferred music improves orientation, attention and executive function. Preferred music produces greater increases in connectivity within the default mode network and boosts activity in prefrontal regions responsible for emotion, cognition and memory processing.^[29]^ Music interventions also play an important role in supporting the emotional, spiritual and social well-being of older adults.^[30]^ By engaging individuals in active or receptive music activities, these interventions can reduce anxiety and agitation while fostering positive mood and opportunities for self-expression.^[31]^ Furthermore, several studies have also highlighted the unique contributions of active music therapy techniques in mild cognitive impairment, demonstrating that engagement in live music‐making activities led by credentialed music therapists can yield additional improvements in working memory, cognitive flexibility, emotional expression, and social connectedness in older adults with MCI.^[32–34]^ Active music therapy interventions (improvisation or re-creating music by singing/playing instruments) have demonstrated tangible social benefits, with group singing circles reducing self-reported loneliness, strengthening peer support networks, and boosting verbal interaction during and after sessions.^[35]^ Cultural differences, including major life transitions, can lead to variability in music-evoked reminiscence, and incorporating culturally familiar elements such as indigenous folk tunes or native-language lyrics enhances emotional engagement and increases session attendance.^[36]^ Indian Classical Music-based interventions have been shown to reduce anxiety, agitation, and cortisol levels in people with cognitive impairment, demonstrating how culturally familiar melodies can enhance engagement and therapeutic outcomes in MCI.^[37]^ In South Korea, a multimodal brain empowerment program that combined music therapy with robot-assisted gait training and brain stimulation elicited significant increases in frontal, temporal, and parietal neural activation, supporting the integration of music into advanced rehabilitation approaches.^[38]^ An Italian scoping review further confirmed that standardized music‐based cognitive stimulation protocols yield improvements in global cognition, verbal fluency, memory, and executive function in early cognitive decline.^[39]^ Moreover, lifelong Carnatic music training among Indian elders was linked to superior visuospatial skills, faster executive task performance, and greater gray matter volumes in key cortical regions, suggesting that sustained musical practice can bolster cognitive reserve and neural resilience.^[40]^

This study has several limitations. Only immediate post-intervention outcomes were assessed, so the long-term effects of music therapy remain unknown. Most included trials were single-center and enrolled relatively small samples, which may introduce selection bias. We only analyzed total MMSE scores; future studies should report domain-specific cognitive outcomes such as memory, attention, and executive control to enable more nuanced evaluations of music therapy effects. Insufficient reporting of intervention details (active vs passive music therapy) precluded any subgroup analysis by therapy modality. Cochrane Risk of Bias tool (ROB 2) assessment identified some concerns in allocation concealment and blinding across several studies, which could affect the robustness of the findings. Finally, the underlying neural and molecular mechanisms were not addressed; we therefore recommend that future research incorporate neuroimaging techniques (e.g., fMRI, positron emission tomography, diffusion tensor imaging, magnetic resonance spectroscopy) and biochemical blood markers (e.g., brain‐derived neurotrophic factor, cortisol) alongside well-designed, multicenter trials with larger cohorts and extended follow-up to validate and deepen these findings.

## 5. Conclusions

Music therapy as a simple and convenient non-drug intervention produced significant improvements in MMSE scores among older adults with mild cognitive impairment, indicating real gains in memory, orientation, attention, and language. However, the optimal overall treatment duration, session length, and weekly frequency have yet to be defined. Large-scale, high-quality trials are needed to establish evidence-based protocols for clinical practice.

## Author contributions

**Conceptualization:** Yi Li, Yu Wang.

**Data curation:** Yi Li, Xiaorong Jiang.

**Formal analysis:** Yi Li, Xiaorong Jiang.

**Investigation:** Meiyu Zhu, Ximei Li.

**Methodology:** Yi Li, Xiaorong Jiang, Meiyu Zhu, Ximei Li.

**Supervision:** Yu Wang.

**Validation:** Yi Li.

**Writing – original draft:** Yi Li, Xiaorong Jiang, Meiyu Zhu, Ximei Li, Yu Wang.

**Writing – review & editing:** Yi Li, Xiaorong Jiang, Meiyu Zhu, Ximei Li, Yu Wang.

## References

[1] Domínguez-Chávez CJ Murrock CJ Salazar-González BC. Mild cognitive impairment: a concept analysis. Nurs Forum. 2019;54:68–76. 30261109 10.1111/nuf.12299

[2] Petersen RC Lopez O Armstrong MJ. Practice guideline update summary: mild cognitive impairment: report of the guideline development, dissemination, and implementation subcommittee of the American Academy of Neurology. Neurology. 2018;90:126–35. 29282327 10.1212/WNL.0000000000004826 PMC5772157

[3] Anderson ND. State of the science on mild cognitive impairment (MCI). CNS Spectr. 2019;24:78–87. 30651152 10.1017/S1092852918001347

[4] Oliveira T Starkweather A Ramesh D. Putative mechanisms of cognitive decline with implications for clinical research and practice. Nurs Forum. 2018;53:271–9. 10.1111/nuf.12247 PMC6438756 29345733

[5] Cheung DSK Lai CKY Wong FKY Leung MCP. The effects of the music-with-movement intervention on the cognitive functions of people with moderate dementia: a randomized controlled trial. Aging Ment Health. 2018;22:306–15. 27819483 10.1080/13607863.2016.1251571

[6] Raglio A Filippi S Bellandi D Stramba-Badiale M. Global music approach to persons with dementia: evidence and practice. Clin Interv Aging. 2014;9:1669–76. 25336931 10.2147/CIA.S71388 PMC4199985

[7] Domínguez-Chávez CJ Murrock CJ Guerrero PIC Salazar-González BC. Music therapy intervention in community-dwelling older adults with mild cognitive impairment: a pilot study. Geriatr Nurs. 2019;40:614–9. 31277962 10.1016/j.gerinurse.2019.06.004

[8] Innes KE Selfe TK Khalsa DS Kandati S. Meditation and music improve memory and cognitive function in adults with subjective cognitive decline: a pilot randomized controlled trial. J Alzheimers Dis. 2017;56:899–916. 28106552 10.3233/JAD-160867 PMC7967907

[9] Page MJ McKenzie JE Bossuyt PM. The PRISMA 2020 statement: an updated guideline for reporting systematic reviews. BMJ. 2021;372:n71. 33782057 10.1136/bmj.n71 PMC8005924

[10] Xiong T. Efficacy Research of Combination of Non-pharmacological Therapy in Elderly Mild Cognitive Impairment. Central South University; 2022.

[11] Miao X. Clinical study on 30 cases of mild cognitive impairment in the elderly treated with Ziwu Liuzhu time-selected five-element music therapy. Jiangsu J Tradit Chin Med. 2020;52:29–31.

[12] Satoh M Ogawa JI Tokita T. Physical exercise with music maintains activities of daily living in patients with dementia: Mihama–Kiho project part 21. J Alzheimers Dis. 2017;57:85–96. 28222531 10.3233/JAD-161217

[13] Satoh M Ogawa J Tokita T. The effects of physical exercise with music on cognitive function of elderly people: Mihama–Kiho project. PLoS One. 2014;9:e95230. 24769624 10.1371/journal.pone.0095230 PMC4000225

[14] Kim HJ Yang Y Oh JG. Effectiveness of a community-based multidomain cognitive intervention program in patients with Alzheimer’s disease. Geriatr Gerontol Int. 2016;16:191–9. 25656505 10.1111/ggi.12453

[15] Kim D. The effects of a recollection-based occupational therapy program of Alzheimer’s disease: a randomized controlled trial. Occup Ther Int. 2020;2020:6305727. 32821251 10.1155/2020/6305727 PMC7416254

[16] Biasutti M Mangiacotti A. Assessing a cognitive music training for older participants: a randomised controlled trial. Int J Geriatr Psychiatry. 2018;33:271–8. 28401595 10.1002/gps.4721

[17] Biasutti M Mangiacotti A. Music training improves depressed mood symptoms in elderly people: a randomized controlled trial. Int J Aging Hum Dev. 2021;92:115–33. 31814419 10.1177/0091415019893988

[18] Islam N Hashem R Gad M. Accuracy of the montreal cognitive assessment tool for detecting mild cognitive impairment: a systematic review and meta-analysis. Alzheimers Dement. 2023;19:3235–43. 36934438 10.1002/alz.13040

[19] González DA Finley JA Patel SES Soble JR. Practical assessment of neuropsychiatric symptoms: updated reliability, validity, and cutoffs for the neuropsychiatric inventory questionnaire. Am J Geriatr Psychiatry. 2025;33:524–34. 39551647 10.1016/j.jagp.2024.10.014 PMC11903187

[20] Cheung DSK Lai CKY Wong FKY Leung MCP. Is music-with-movement intervention better than music listening and social activities in alleviating agitation of people with moderate dementia? A randomized controlled trial. Dementia (London). 2020;19:1413–25. 30235949 10.1177/1471301218800195

[21] Talwar P Kushwaha S Chaturvedi M Mahajan V. Systematic review of different neuroimaging correlates in mild cognitive impairment and Alzheimer’s disease. Clin Neuroradiol. 2021;31:953–67. 34297137 10.1007/s00062-021-01057-7

[22] Alluri V Toiviainen P Jääskeläinen IP Glerean E Sams M Brattico E. Large-scale brain networks emerge from dynamic processing of musical timbre, key and rhythm. Neuroimage. 2012;59:3677–89. 22116038 10.1016/j.neuroimage.2011.11.019

[23] Kasdan AV Burgess AN Pizzagalli F. Identifying a brain network for musical rhythm: a functional neuroimaging meta-analysis and systematic review. Neurosci Biobehav Rev. 2022;136:104588. 35259422 10.1016/j.neubiorev.2022.104588 PMC9195154

[24] Wei Y Gan L Huang X. A review of research on the neurocognition for timbre perception. Front Psychol. 2022;13:869475. 35422736 10.3389/fpsyg.2022.869475 PMC9001888

[25] Li CW Guo FY Tsai CG. Predictive processing, cognitive control, and tonality stability of music: an fMRI study of chromatic harmony. Brain Cogn. 2021;151:105751. 33991840 10.1016/j.bandc.2021.105751

[26] Molendijk ML Spinhoven P Polak M Bus BA Penninx BW Elzinga BM. Serum BDNF concentrations as peripheral manifestations of depression: evidence from a systematic review and meta-analyses on 179 associations (N=9484). Mol Psychiatry. 2014;19:791–800. 23958957 10.1038/mp.2013.105

[27] Sun Y Shi X Ohm M Korte M Zagrebelsky M. Deciphering genetic causality between plasma BDNF and 91 circulating inflammatory proteins through bidirectional Mendelian randomization. Sci Rep. 2025;15:10312. 40133606 10.1038/s41598-025-95546-1 PMC11937598

[28] Koelsch S. Brain correlates of music-evoked emotions. Nat Rev Neurosci. 2014;15:170–80. 24552785 10.1038/nrn3666

[29] Pérez-Ros P Cubero-Plazas L Mejías-Serrano T Cunha C Martínez-Arnau FM. Preferred music listening intervention in nursing home residents with cognitive impairment: a randomized intervention study. J Alzheimers Dis. 2019;70:433–42. 31177232 10.3233/JAD-190361

[30] Qiu L Zhong Y Xie Q. Multi-modal integration of EEG-fNIRS for characterization of brain activity evoked by preferred music. Front Neurorobot. 2022;16:823435. 35173597 10.3389/fnbot.2022.823435 PMC8841473

[31] Hilliard RE. A post-hoc analysis of music therapy services for residents in nursing homes receiving hospice care. J Music Ther. 2004;41:266–81. 15762834 10.1093/jmt/41.4.266

[32] Ghilain M Hobeika L Lesaffre M. Does a live performance impact synchronization to musical rhythm in cognitively impaired elderly? J Alzheimers Dis. 2020;78:939–49. 33104027 10.3233/JAD-200521

[33] Raglio A Bellandi D Baiardi P. Effect of active music therapy and individualized listening to music on dementia: a multicenter randomized controlled trial. J Am Geriatr Soc. 2015;63:1534–9. 26289682 10.1111/jgs.13558

[34] Raglio A Bellandi D Baiardi P Gianotti M Ubezio MC Granieri E. Listening to music and active music therapy in behavioral disturbances in dementia: a crossover study. J Am Geriatr Soc. 2013;61:645–7. 23581919 10.1111/jgs.12187

[35] Davidson JW McNamara B Rosenwax L Lange A Jenkins S Lewin G. Evaluating the potential of group singing to enhance the well-being of older people. Australas J Ageing. 2014;33:99–104. 24520864 10.1111/j.1741-6612.2012.00645.x

[36] Von Fritschen C Dos Santos A. Perspectives on music healing by traditional healers and music therapists. Music Ther. Perspect.. 2023;41:152–8.

[37] Ghosh A Jain S Issac TG. Indian music: a future in dementia care Indian music and dementia. Indian J Psychol Med. 2024;46:187–9. 38725714 10.1177/02537176231201562 PMC11076945

[38] Oh W Park H Hallett M You JSH. The effectiveness of a multimodal brain empowerment program in mild cognitive impairment: a single-blind, quasi-randomized experimental study. J Clin Med. 2023;12:4895. 37568297 10.3390/jcm12154895 PMC10419895

[39] Raglio A Figini C Bencivenni A Grossi F Boschetti F Manera MR. Cognitive stimulation with music in older adults with cognitive impairment: a scoping review. Brain Sci. 2024;14:842. 39199533 10.3390/brainsci14080842 PMC11352551

[40] Ghosh A Singh S Monisha S Jagtap T Issac TG. Music and the aging brain - exploring the role of long-term Carnatic music training on cognition and gray matter volumes. J Neurosci Rural Pract. 2024;15:327–33. 38746502 10.25259/JNRP_605_2023 PMC11090532

